# Adaptor Protein Complexes AP-1 and AP-3 Are Required by the HHV-7 Immunoevasin U21 for Rerouting of Class I MHC Molecules to the Lysosomal Compartment

**DOI:** 10.1371/journal.pone.0099139

**Published:** 2014-06-05

**Authors:** Lisa A. Kimpler, Nicole L. Glosson, Deanna Downs, Patrick Gonyo, Nathan A. May, Amy W. Hudson

**Affiliations:** Department of Microbiology and Molecular Genetics, Medical College of Wisconsin, Milwaukee, Wisconsin, United States of America; Purdue University, United States of America

## Abstract

The human herpesvirus-7 (HHV-7) U21 gene product binds to class I major histocompatibility complex (MHC) molecules and reroutes them to a lysosomal compartment. Trafficking of integral membrane proteins to lysosomes is mediated through cytoplasmic sorting signals that recruit heterotetrameric clathrin adaptor protein (AP) complexes, which in turn mediate protein sorting in post-Golgi vesicular transport. Since U21 can mediate rerouting of class I molecules to lysosomes even when lacking its cytoplasmic tail, we hypothesize the existence of a cellular protein that contains the lysosomal sorting information required to escort class I molecules to the lysosomal compartment. If such a protein exists, we expect that it might recruit clathrin adaptor protein complexes as a means of lysosomal sorting. Here we describe experiments demonstrating that the μ adaptins from AP-1 and AP-3 are involved in U21-mediated trafficking of class I molecules to lysosomes. These experiments support the idea that a cellular protein(s) is necessary for U21-mediated lysosomal sorting of class I molecules. We also examine the impact of transient *versus* chronic knockdown of these adaptor protein complexes, and show that the few remaining μ subunits in the cells are eventually able to reroute class I molecules to lysosomes.

## Introduction

Like all other herpesviruses, HHV-6 and -7 remain latent or establish persistent infections. To do so, they must avoid detection and elimination by the immune system. Notably, most of the herpesviruses thus far examined employ the strategy of interfering with viral antigen presentation to cytotoxic T lymphocytes (CTLs) (for review, see [1]). Some herpesviral proteins interfere with proteolysis of antigens or peptide transport into the ER [2]–[4]. Others retain class I molecules in the ER, mediate their destruction through ER-associated degradation, enhance the internalization of class I molecules, or divert class I molecules to lysosomes for degradation [5]–[14]. Judging from the number and molecular diversity of these strategies, the removal of class I MHC-peptide complexes from the cell surface must be evolutionarily advantageous to these viruses, likely as a means of escaping immune detection.

HHV-6 and -7 encode a type I membrane glycoprotein, U21, that specifically reroutes newly-synthesized, properly-folded class I MHC molecules to the lysosomal compartment for degradation [7], [8]. Rather than encode an entirely novel mechanism of lysosomal trafficking, we hypothesize that this single viral protein is more likely to usurp a pre-existing cellular lysosomal sorting pathway for its own benefit. In postulating a mechanism by which U21 mediates lysosomal sorting, we first noted that the trafficking of integral membrane proteins to the lysosomal pathway is generally mediated by proteins that recognize tyrosine- or di-leucine-based cytoplasmic sorting signals. The lysosomal membrane proteins lamp-1 and lamp-2, for example, contain a YxxΦ sorting motif, characterized by an essential tyrosine residue and a bulky hydrophobic amino acid, while limp-2 contains a di-leucine-based sorting motif (for review, see [15]). The Y-based and di-leucine sorting motifs are recognized by clathrin adaptor protein complexes AP-1 and AP-3 (for review, see [15]). Internalization of membrane proteins from the plasma membrane is mediated by AP-2, while some clathrin-independent TGN-endosomal trafficking may be mediated by AP-4 [16], [17].

A membrane protein can take two routes to reach lysosomes, “direct” or “indirect” [18], [19]. The “direct” route involves trafficking of a membrane protein to lysosomes from the TGN without visiting the cell surface. For example, in the direct pathway, a membrane protein can travel from the TGN to a late endosomal compartment, followed by subsequent transport to lysosomes, or can traffic directly from the TGN to lysosomes. The “indirect” route involves trafficking of a membrane protein from the ER to the Golgi/TGN, and then to the cell surface, where it is internalized and then travels through the endocytic system, eventually reaching lysosomes. We do not know which of these routes is employed by U21 as it escorts class I MHC molecules to the lysosomal compartment. We originally hypothesized that if U21 travels to lysosomes via the indirect pathway, we should be able to detect it at the cell surface, using surface biotinylation. Because we have been unable to detect U21 on the cell surface, we favor the possibility that U21 uses the direct route to lysosomes. However, we cannot rule out the possibility that U21’s appearance at the cell surface is fleeting, or that U21’s N-linked glycans prevent its surface-biotinylation. Since AP-2 mediates internalization from the cell surface, involvement of AP-2 in U21-mediated sorting of class I MHC molecules to lysosomes would suggest that U21 uses the indirect pathway. Likewise, involvement of AP-1, AP-3 or AP-4, in U21-mediated class I diversion would implicate the direct route.

In the simplest model for U21-mediated trafficking of class I MHC molecules to lysosomes, U21 would contain a sorting signal in its cytoplasmic tail. This model is exemplified by the murine cytomegalovirus (MCMV) m06 gene product: MCMV m06, which encodes a glycoprotein of 48 kDa (gp48) that binds to class I MHC molecules, contains a di-leucine sorting signal in its cytoplasmic tail that recruits AP-1 and AP-3 clathrin adaptor complexes [11]. These adaptor complexes mediate sorting of MCMV gp48 and class I MHC molecules to the endolysosomal compartment [20].

To determine whether HHV-7 U21 employs a mechanism similar to that of MCMV gp48, we deleted the cytoplasmic tail of U21. Surprisingly, we found that U21 can affect the trafficking of class I molecules even when lacking its cytoplasmic tail, and indeed, even when U21 is expressed as a soluble secreted protein that lacks both its single transmembrane domain and cytoplasmic tail [21], [22]. Thus, if U21 makes use of a conventional cytoplasmic sorting signal, the signal is not contained within U21. [22] We therefore favor the idea that a cellular protein(s) associates with the U21/class I MHC complex, and that this protein contains the cytoplasmic sorting signal necessary to reroute the class I MHC-U21 complexes. To explore this possibility, we reasoned that if U21 utilizes a cellular protein X that relies upon the clathrin adaptor proteins for direction, then knockdown of the essential clathrin adaptor complexes should impair U21-mediated trafficking of class I MHC molecules to lysosomes. Herein we examine the U21-mediated trafficking of class I MHC molecules after RNAi-mediated depletion of the adaptor protein complexes AP-1, -2, -3, and -4.

## Materials and Methods

### Cell Lines

293T cells (kindly provided by Dr. Peter Howley, Harvard Medical School) (American Type Culture Collection, Manassas, VA) and U373-MG astrocytoma cells (kindly provided by Dr. Hidde Ploegh, Harvard Medical School) (originally purchased from ATCC) were cultured in Dulbecco’s modified Eagle medium (DMEM) supplemented with 5% fetal bovine serum and 5% newborn calf serum in the absence or presence of puromycin [375 ng/ml final] (Sigma-Aldrich, St. Louis, MO, USA), or geneticin (G418) [400 ng/ml final] (Gibco, Grand Island, NY, USA). Generation of stably-expressing U373-U21 cells was carried out previously via retrovirus-mediated gene transfer, as described [7], [8].

### Antibodies

The following monoclonal antibodies (mAb) were used: mAb W6/32, reactive with assembled, β_2_m-associated HLA-A, -B, or -C molecules (generously provided by H. Ploegh, Whitehead Institute, Cambridge, MA); FITC-conjugated W6/32 (eBioscience, Inc., San Diego, CA); H4B4, directed against lamp2 (generously provided by T. August, Johns Hopkins Medical School, Baltimore, MD) [23]; 100/3, directed against the γ1 subunit of AP-1 (generously provided by T. Kirchhausen, Harvard University, Cambridge, MA); AP.6 and MA3-061 to α-adaptin (generously provided by P. DeCamilli, Yale University, New Haven, CT). The following rabbit polyclonal antibodies (pAb) were used: HA.11, directed against the HA epitope tag (Covance, SanDiego, CA); RY-1, to μ1A (generously provided by L. Traub, University of Pittsburgh, Pittsburgh, PA) [24]; antiserum directed against μ1B, generously provided by Dr. H. Fölsch (Northwestern University, Evanston, IL) [25]; MCW50, raised against the cytoplasmic tail of HHV-7 U21 [22]. Alexa-Fluor 488, 594, and 647-conjugated secondary antibodies were used in immunoflourescence studies (Molecular Probes, Eugene, OR). In some instances, Alexa 594-conjugated Fab fragments (Zenon, Molecular Probes) were used to label lamp2 mAbs. A phycoerythrin (PE)-conjugated anti-HLA-A,B,C mAb (BD Pharmingen, San Jose, CA) was used in flow cytometry experiments. Horseradish peroxidase (HRP)-conjugated monoclonal and polyclonal secondary antibodies (Bio-Rad, Hercules, CA) were used for immunoblotting.

### Plasmids and shRNA Constructs

gp48 was amplified from an MCMV BAC clone (generously provided by Dr. Caroline Kulesza, [26]) and an HA tag was fused in-frame to the C-terminus of MCMV gp48 using the primers 5′- AAAACTCGAGGCCACCATGCCCAGTTGGAGCGAT-3′ and 5′- AAAAGGATCCTCATGCGTAGTCTGGTACGTCGTATGGGTATTTGGTAAGCAAGGGGGA-3′. HA-tagged and non-tagged gp48 were subcloned into the pPM lentiviral vector (pHAGE-puro-MCS) [27], which was used to generate gp48- and gp48-HA-expressing cell lines, as described previously [28]. The shRNA-expressing lentiviral vector pLentiLox 3.7 (pLL3.7) was used to generate RNAi constructs targeting the μ subunits of AP-1 and AP-3, and the α subunit of AP-2 [29]. For each AP subunit, a 19-nucleotide target sequence was chosen based on previous studies and cloned into the MCS of pLL3.7 via XhoI and HpaI. The target sequences used in this study are as follows: GTCCGTTTCATGTGGATCA (for AP-1μ), GCCAGTCTGTCTGTGATTA (for AP-3μ) [30], and GAGCATGTGCACGCTGGCCA (for AP-2α) [31], and AAGTCTCGTTTCTCTGCTCTG (for AP-4μ) [30]. shRNA-encoding lentiviral vectors were purchased from Sigma, and used to target AP-1γ (ref seq NM_001128), AP-2μ (ref seq NM_004068), and AP-3δ (ref seq NM_003938) (Sigma-Aldrich, St. Louis, MO). Following lentiviral transduction, cells were selected in puromycin. The FLAG-tagged AP-3μ construct resistant to shRNA μ3 was generated using overlap-PCR mutagenesis. The sequence for μ3 corresponding to nucleotide positions 74–92 was mutated to 5′-AG**T**CA**A**TC**A**GT**G**TG**C**GATTATTTCTT, leaving the amino acid sequence unchanged.

### Small Interfering RNA Transfection

The following previously published siRNA targets were purchased from Dharmacon (Lafayette, CO) and used for siRNA knockdown experiments: AAGGCAUCAAGUAUCGGAAGA (for AP-1μ), AAGGAGAACAGUUCUUGCGGC (for AP-2α), AAGGAGAACAGUUCUUGCGGC (for AP-3μ), and AAGTCTCGTTTCTCTGCTCTG (for AP-4μ). siRNA transfection of U373 and U2-expressing cells was carried out using oligofectamine, according to the manufacturer’s protocol (Invitrogen, Carlsbad, CA).

### Immunoblots

Cells were lysed in NP-40 lysis buffer containing 1% NP-40, 150 mM NaCl, 50 mM Tris-HCl pH 7.4, and 5 mM MgCl_2_. Protein concentrations in cell lysates were assayed in triplicate using the Pierce BCA protein assay kit (Pierce, Rockford, IL). Proteins were separated using SDS-PAGE, transferred to BA-85 nitrocellulose membrane (Whatman, Florham Park, NJ), and probed with the indicated primary Abs, followed by an HRP-conjugated secondary Ab (Bio-Rad). Equal loading of samples was verified using Ponceau S (Sigma-Aldrich) to stain the nitrocellulose membrane. Polypeptides were visualized using the Pierce Supersignal chemiluminescence reagents (Pierce, Rockford, IL) and in some cases quantitated with an Alpha Imager.

### Immunofluorescence Microscopy

Cells on coverslips were washed with PBS and fixed with 4% paraformaldehyde for 20 min, permeabilized with 0.5% saponin in PBS and 3% BSA, incubated with primary antibodies for 30 min, washed, and followed by Alexa 488-, 594-, or 647-conjugated secondary antibodies. In some instances, if two monoclonal antibodies were used, after incubation in secondary antibody, cells were washed and incubated in FITC-conjugated W6/32 tertiary antibody.

### Flow Cytometry

Cells were removed from the plates, washed with ice-cold PBS, incubated with PE anti-HLA-A,B,C in PBS and 1% BSA for 30 min on ice, washed thoroughly, and resuspended in ice-cold PBS and 1% BSA. Cell surface expression of class l MHC molecules was then evaluated with a Beckman FACScalibur flow cytometer or with a Guava EasyCyte Mini flow cytometer (Millipore, Billerica, MA).

## Results

### Depletion of AP-2 does not Affect U21-mediated Diversion of Class I MHC Molecules

In selecting a strategy for depletion of the APs, we chose small hairpin (sh)RNA-mediated knockdown.

**Figure 1:**
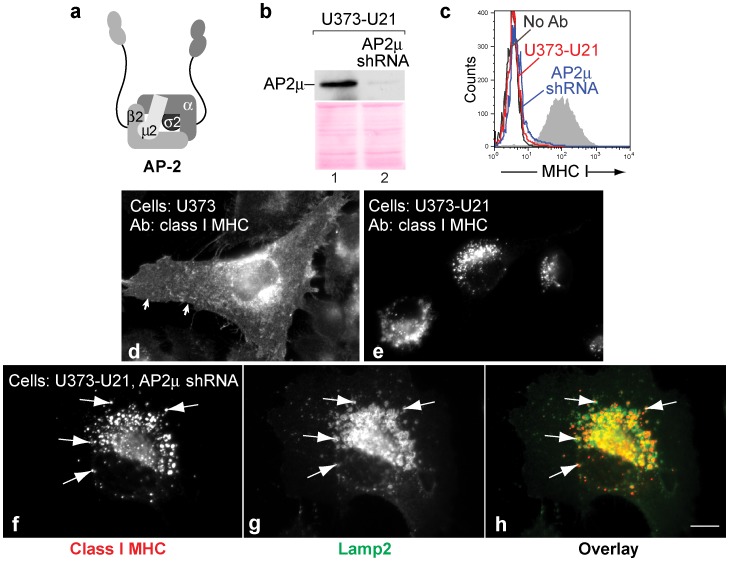
U21 can reroute class I MHC molecules in the absence of AP2µ. a) Schematic representation of the AP-2 complex (redrawn from [15]). b) AP2µ immunoblot of lysates from U373-U21 cells before and 5 days after introduction of AP-2µ shRNA. The Ponceau S stained nitrocellulose is shown beneath the immunoblot as a loading control. c) Flow cytometric analysis of class I MHC molecules on the cell surface of U373 or U373-U21 cells, 5 days after introduction of AP-2µ shRNA. Cell lines − (red) and + (blue) AP-2µ shRNA are indicated. d,e) U373 and U373-U21 cells or AP2µ shRNA-expressing U373 and U373-U21 cells were labeled with W6/32, directed against properly-folded class I MHC molecules, as indicated. Arrows in panel d point to the plasma membrane. f,g) U373-U21 cells were double-labeled with W6/32 and anti-lamp2 Arrows point to specific puncta that overlap. h). The images are shown overlayed in (h), with class I molecules in red, and lamp2 in green, as indicated. Cells are shown at the same magnification. Scale bar = 10 µm.

In general, depletion of any one of the four AP subunits reduces the possibility of heterotetramer formation, and thereby affects AP-mediated sorting (see schematic, Figure 1a, [31]–[33]. Using lentivirus-mediated gene transfer of an shRNA to the µ subunit of AP-2, (μ2) we reduced μ2 levels in U21-expressing U373 cells by 98% (Figure 1b). To assess the effect of AP-2 depletion upon U21’s ability to reroute class I MHC molecules to lysosomes, we examined the cells for surface expression of class I molecules using flow cytometry. Control U373 cells exhibit labeling of class I MHC molecules at the cell surface (Figure 1c, shaded trace). When U21 is expressed in U373 cells, surface levels of class I MHC molecules are dramatically reduced (Figure 1c, compare red to black traces). When μ2 was depleted from U21-expressing cells, surface levels of class I molecules remained low, strongly suggesting that AP-2 does not play a role in mediating trafficking of class I molecules to the plasma membrane (Figure 1c, blue trace).

Immunolocalization experiments mirrored the flow cytometric analysis. When we examine the localization of class I MHC molecules in U373 cells, class I molecules are largely present on the cell surface (Figure 1, panel d), and in U21-expressing cells, class I molecules are localized to a lysosomal compartment (Figure 1, panel e, [8]). In the U21 cells depleted of μ2, class I MHC molecules are present in similar perinuclear punctae (Figure 1, compare panels e and f). To be sure that μ2-depletion resulted in the trafficking of class I molecules to lysosomes, and not to a different compartment, we performed double-label immunofluorescence colocalization with the lysosomal membrane protein marker lamp2. In the μ2-depleted U21 cells, we observed colocalization between lamp2 and class I molecules (Figure 1, panels f-h). These results again suggest that the AP-2 adaptor complex does not participate in U21-mediated diversion of class I MHC molecules to the lysosomal compartment.

### Depletion of AP-1 Affects U21-mediated Rerouting of Class I MHC Molecules

**Figure 2:**
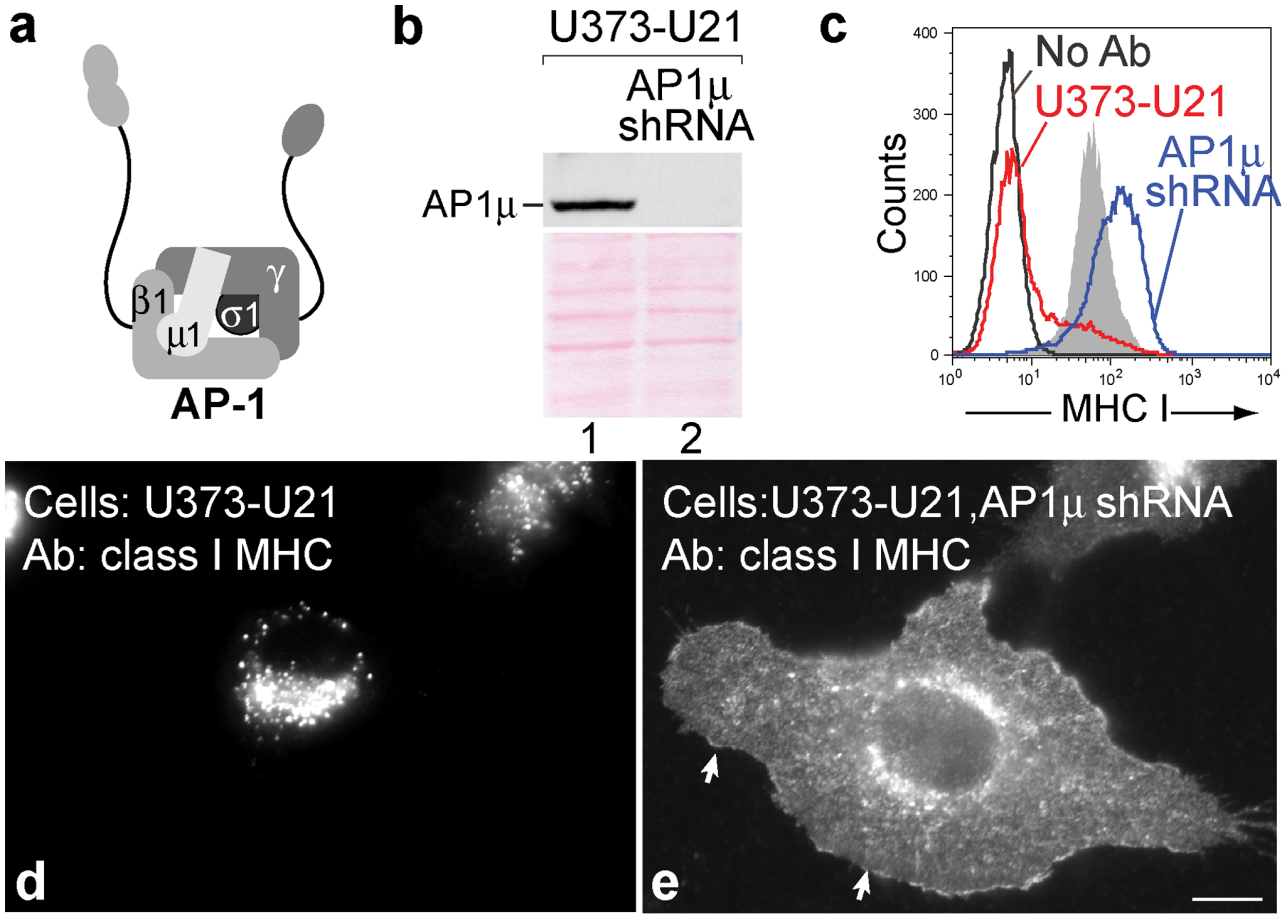
U21 does not reroute class I MHC molecules in the absence of AP1µ. a) Schematic representation of the AP-1 complex (redrawn from [15]). b) AP-1µ immunoblot of lysates from U373-U21 cells before and 5 days after introduction of AP1µ shRNA. The Ponceau S stained nitrocellulose is shown beneath the immunoblot as a loading control c) Flow cytometric analysis of class I MHC molecules on the cell surface of U373 or U373-U21 cells, 5 days after introduction of AP-1µ shRNA. Cell lines − (red) and + (blue) AP-1µ shRNA are indicated. d,e) U373 and U373-U21 cells or AP-1µ shRNA-expressing U373 and U373-U21 cells were labeled with W6/32, as indicated, 5 days after introduction of AP-1µ shRNA. Arrows in panel e point to the plasma membrane. Scale bar = 10 µm.

We next assessed RNAi-mediated reduction of AP-1. Using lentivirus-mediated gene transfer of an shRNA to the µ1 subunit of AP-1, we reduced μ1 in U21-expressing U373 cells to an undetectable level (Figure 2b). To assess the effect of AP-1 depletion upon U21’s ability to reroute class I MHC molecules to lysosomes, we again examined the cell surface expression and immunolocalization of class I molecules in cells depleted of μ1. When μ1 was depleted in U21 cells, surface levels of class I molecules increased from undetectable to levels comparable to control U373 cells, strongly suggesting that AP-1 is involved in U21-mediating trafficking of class I molecules to the lysosomes (Figure 2c, compare blue and shaded traces). Immunofluorescence experiments designed to examine the subcellular localization of class I molecules mirrored the cytometric analysis, showing a striking increase in the surface localization of class I molecules after μ1 depletion (Figure 2, compare panels d and e). These results suggest that AP-1 participates in U21-mediated diversion of class I MHC molecules to the lysosomal compartment.

### Depletion of AP-3 Affects U21-mediated Rerouting of Class I MHC Molecules

**Figure 3:**
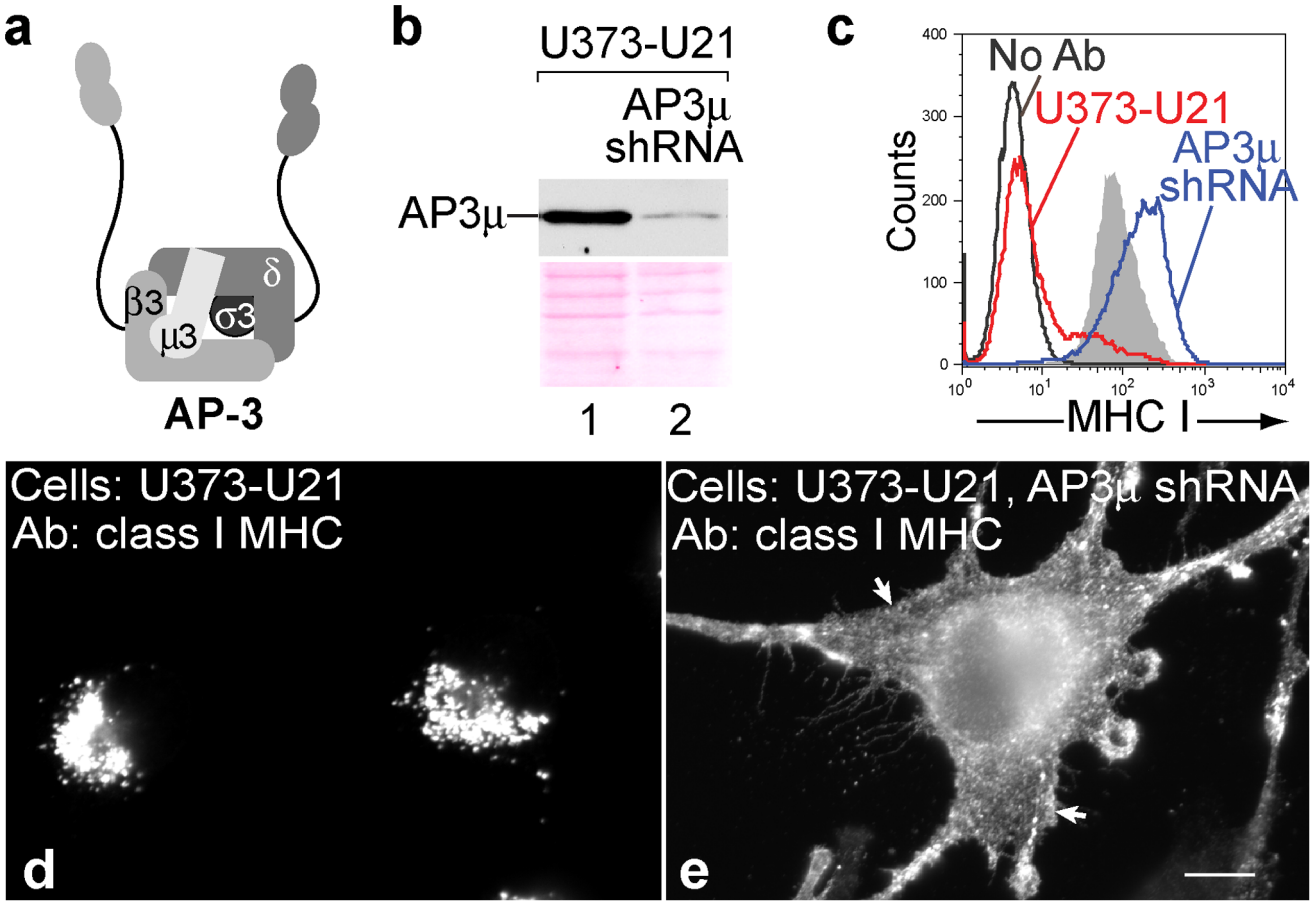
U21 does not reroute class I MHC molecules in the absence of AP3µ. a) Schematic representation of the AP-3 complex (redrawn from [15]). b) AP-3µ immunoblot of lysates from U373-U21 cells before and 5 days after introduction of AP-3µ shRNA. The Ponceau S stained nitrocellulose is shown beneath the immunoblot as a loading control c) Flow cytometric analysis of class I MHC molecules on the cell surface of U373 or U373-U21 cells, 5 days after introduction of AP-3µ shRNA. Cell lines − (red) and + (blue) AP-3µ shRNA are indicated. d,e) U373 and U373-U21 cells or AP-3µ shRNA-expressing U373 and U373-U21 cells were labeled with W6/32, as indicated, 5 days after introduction of AP-3µ shRNA. Arrows in panel e point to the plasma membrane. Scale bar = 10 µm.

We also examined the potential role of AP-3 in U21-mediated trafficking of class I MHC molecules to lysosomes. Just as for AP-1, we used shRNA to reduce the levels of the AP-3 μ3 subunit, reducing μ3 in U21-expressing cells by 93% (Figure 3b). Again, the redistribution of class I MHC molecules to the cell surface in U21 cells was increased to levels comparable to control U373 cells, suggesting that AP-3 is also involved in mediating trafficking of class I molecules to the lysosomal compartment (Figure 3c, compare blue and shaded traces). Immunofluorescence experiments performed to examine the subcellular localization of class I molecules again mirrored the cytometric analysis, showing a striking increase in the surface localization of class I molecules after μ3 depletion (Figure 3, compare panels d and e), suggesting that AP-3, too, participates in U21-mediated diversion of class I MHC molecules to the lysosomal compartment. Of note, AP-4 μ4 depletion had no effect upon U21-mediated trafficking of class I molecules (data not shown).

### siRNA-mediated Depletion Parallels shRNA Depletion

**Figure 4:**
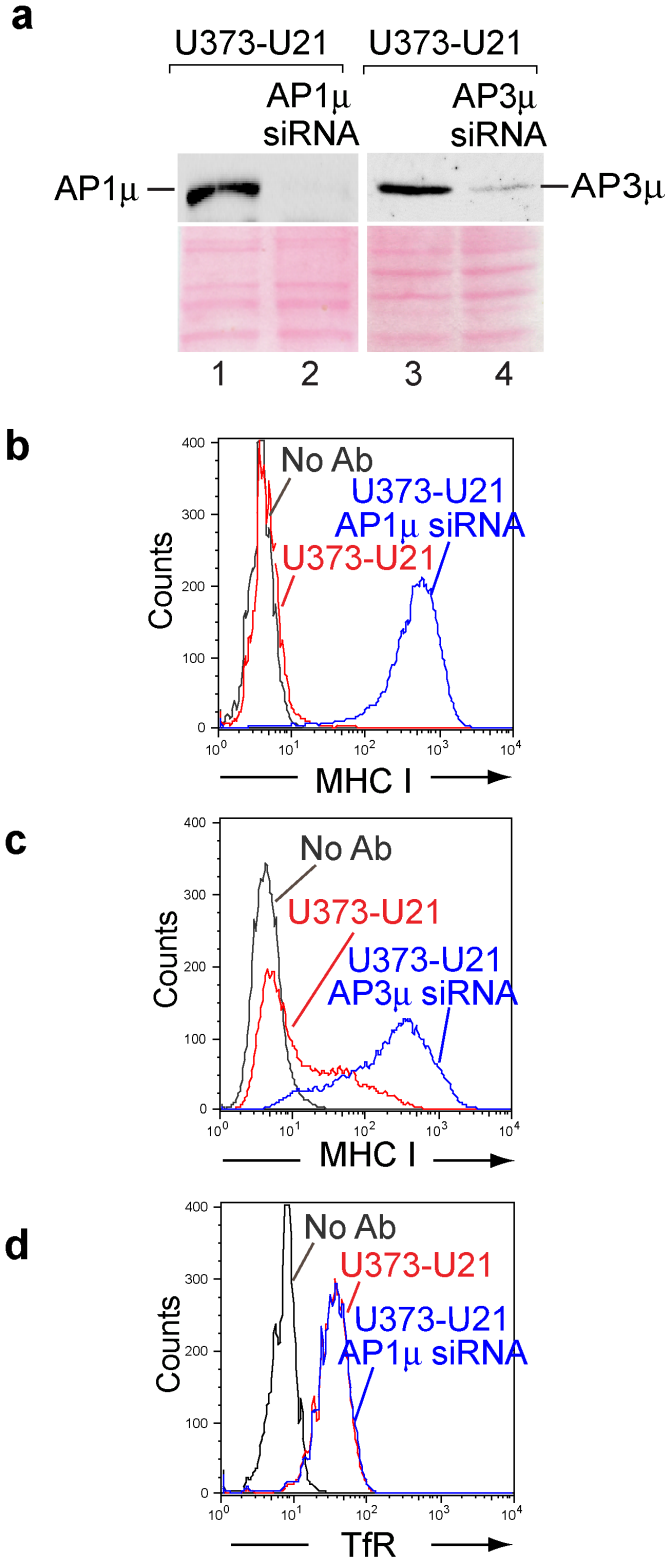
siRNA-mediated depletion of AP-1 and AP-3. a) anti-AP-1μ or anti-AP-3μ immunoblot analysis of lysates from U373-U21 cells −/+ siRNA mediated depletion, as indicated. The Ponceau S stained nitrocellulose is shown beneath the immunoblots as a loading control. b,c) Flow cytometric analysis of class I MHC molecules on the cell surface of U373-U21 cells, −/+ AP-1μ or AP-3µ siRNA. Cell lines − (red traces) and + (blue traces) AP-1μ (b) or AP-3µ (c) siRNA are indicated. d) Cell surface levels of the transferrin receptor (TfR) in U373-U21 cells −/+ siRNA-mediated depletion of AP1μ, as indicated.

Because we were concerned that the μ1 and μ3 shRNA-containing lentiviruses might elicit off-target effects resulting in class I MHC relocalization, we also employed small interfering RNAs (siRNAs) to deplete AP-1 and AP-3. We selected siRNA sequences that were targeted to different regions of the μ1 and μ3 mRNAs than the shRNAs. These siRNAs had been successfully employed in other studies to reduce AP-1 and AP-3 levels [30], [31], [34], [35]. The siRNAs were effective in depleting the μ1 to below the limit of detection, and depleted the μ3 subunit by 97% (Figure 4a, compare lanes 1 and 2, 3 and 4). siRNA-mediated depletion of μ1 and μ3 identically mirrored our shRNA-mediated depletion experiments, resulting in the reappearance of class I MHC molecules at the cell surface (Figure 4b and c, compare red and blue traces). To ensure that depletion of adaptor complex did not have more far-reaching effects upon other integral membrane proteins, we also examined the cell surface expression of the transferrin receptor (TfR) on U21-expressing cells depleted of μ1. TfR is unaffected in cells expressing U21 [8], [36], and as shown previously, transferrin receptor was unaffected by depletion of μ1 (Figure 4d) [30], [37]. Likewise, depletion of μ3 subunits in U21-expressing cells had no apparent effect upon TfR distribution (data not shown, [30], [37]).

### Expression of shRNA-resistant AP-3μ Rescues Effect of Knockdown

**Figure 5:**
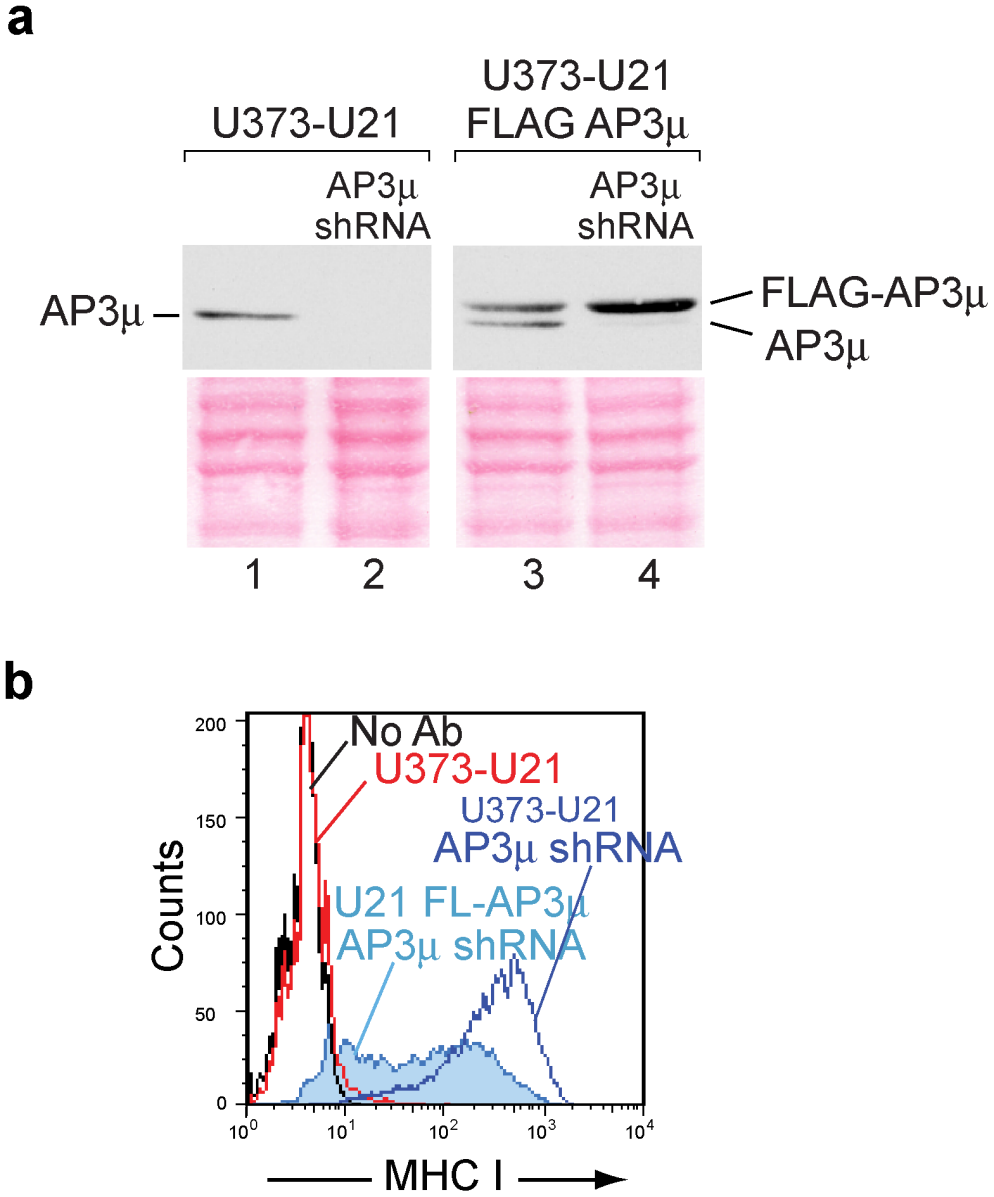
Rescue of AP-3 depletion. a) anti-AP-3μ immunoblot analysis of lysates from U373-U21 cells (lanes 1,2) or U373-U21 cells expressing FLAG-AP-3μ^ rescue^ (lanes 3,4), −/+ AP-3μ shRNA, as indicated. The Ponceau S stained nitrocellulose is shown beneath the immunoblots as a loading control. b) Flow cytometric analysis of surface class I MHC molecules on U373-U21 or U373-U21 cells expressing FLAG-AP-3 µ^rescue^, −/+ AP-3μ shRNA depletion, as indicated.

As another means to demonstrate the validity of our shRNA-mediated reduction of AP complexes, we stably expressed an shRNA-resistant FLAG-tagged μ3 in U373-U21 cells (Figure 5a, compare lane 3 to lane 1). We then used shRNA to deplete endogenous μ3 (Figure 5a, lanes 2 and 4). The presence of FLAG-tagged μ3^rescue^ clearly impaired the shRNA-induced class I MHC relocalization to the cell surface (Figure 5b). While shRNA-mediated depletion of μ3 resulted in a population of cells expressing high surface levels of class I molecules (Figure 5b, blue trace), the presence of FLAG-tagged μ3^rescue^ affected the ability of the μ3 shRNA to induce a dramatic increase in surface class I molecules (Figure 5b, light blue shaded trace). Instead, the cells distributed bimodally, suggesting that the FLAG-μ3^rescue^ subunits were expressed at varying levels within the stable pooled population of μ3^rescue^ cells. There is evidence to suggest that an N-terminal epitope tag does not hamper the function of the μ2 subunit [38], but it remains possible that the presence of the N-terminal FLAG tag impairs the function of the μ3 subunit, resulting in only partial rescue. In sum, however, expression of an μ3^rescue^ subunit had a clear effect upon the ability of the AP3μ shRNA to cause redistribution of class I molecules to the cell surface. Altogether, these results alleviated our concerns about off-target effects, and suggest that AP-1 and AP-3 participate in U21-mediated diversion of class I MHC molecules to the lysosomal compartment.

### Tailless U21 is also Affected by Depletion of Adaptor Complexes

**Figure 6:**
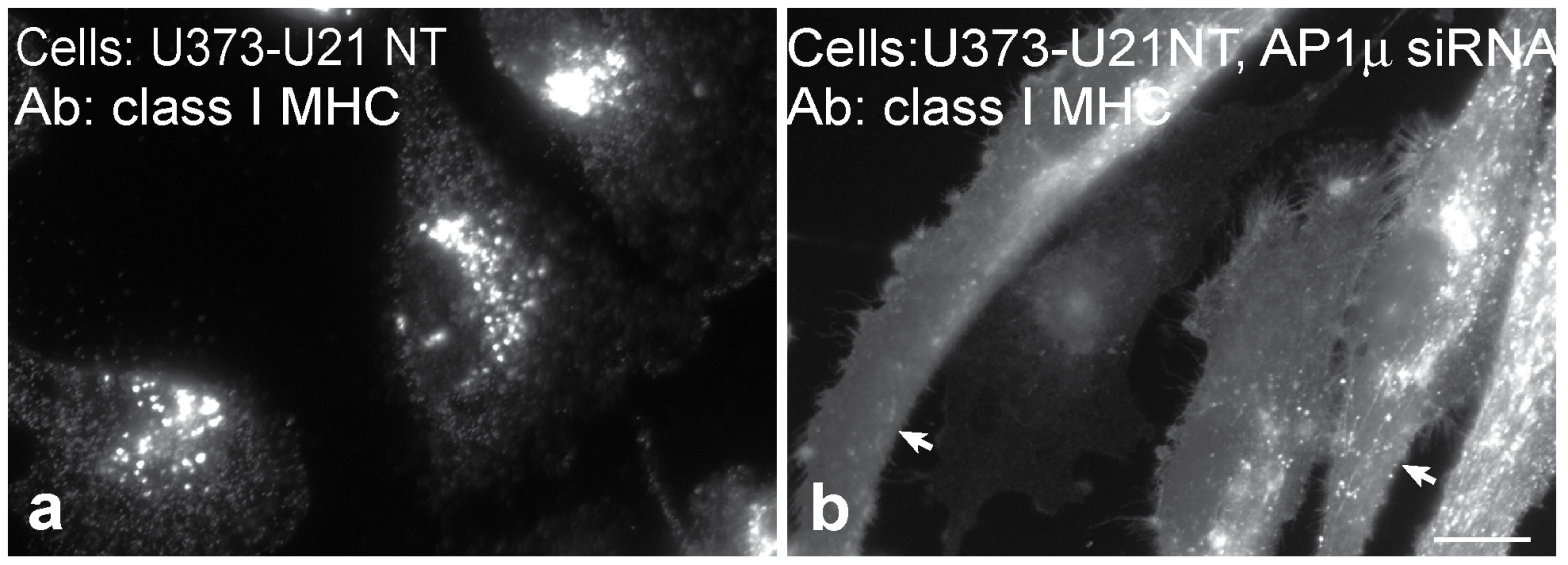
Tailless U21 is affected by depletion of adaptor complexes. Immunofluorescence analysis of class I MHC molecules in tailless-U21-expressing cells (U21NT), − (a) and + (b) AP-1μ shRNA expression, as indicated. Arrows in panel b point to the plasma membrane. Scale bar = 10 µm.

U21 can reroute class I MHC molecules even when U21 lacks its cytoplasmic tail [21], [22], thus the involvement of cytoplasmic AP-1 and AP-3 in U21-mediated diversion of class I molecules invokes a third protein that possesses a cytoplasmic domain to recruit these adaptor complexes. To rule out the possibility that the cytoplasmic tail of U21 is at all responsible for AP recruitment, we performed depletion of AP-1μ in cells expressing tailless U21. As we have shown previously, class I MHC molecules in cells expressing tailless U21 are diverted to the lysosomal compartment (Figure 6, panel a [21]). After siRNA-mediated depletion of μ1 from cells expressing tailless U21, class I MHC molecules redistributed to the cell surface (Figure 6, panel b), demonstrating that AP-1 does not require the cytoplasmic tail of U21 to sort class I molecules to lysosomes, and suggesting the existence of a cellular protein X that is dependent on AP-1 and AP-3 to mediate rerouting of U21 and class I molecules to the lysosomal compartment.

### Class I Molecules Return to the Plasma Membrane after Time in Culture

**Figure 7:**
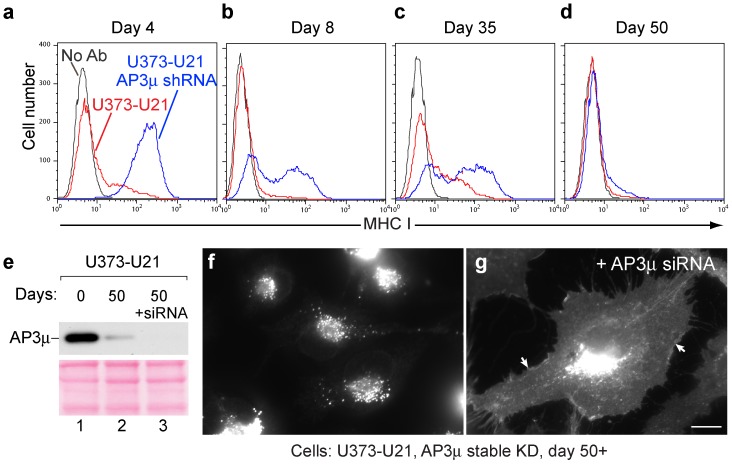
AP-3μ shRNA depletion becomes ineffective over time. a–d) Flow cytometric analysis of class I MHC molecules on the cell surface of U373-U21 cells a) 4, b) 8, c) 35, and d) 50 days after introduction of AP-3µ shRNA. Surface levels of class I MHC, − (red) and + (blue) AP-3µ shRNA are indicated. e) Immunoblot analysis of lysates from U373-U21-AP-3μ shRNA-expressing cells −/+ introduction of AP-3µ siRNA, after 0 and 50 days in culture, as indicated. The Ponceau S stained nitrocellulose is shown beneath the immunoblot as a loading control. f and g) Immunofluorescence analysis of shRNA-depleted U373-U21 cells after 50 days in culture, −/+ AP-3μ siRNA treatment, as indicated. Arrows in panel g point to the plasma membrane. Scale bar = 10 µm.

To generate a large enough population of AP-knockdown cells from which to perform immunoprecipitations, we chose to use an shRNA vector containing a selectable puromycin resistance cassette, with which we could create stable cell lines lacking AP complexes. We cultured our U21 and shRNA-expressing μ1- and μ3-depleted cells to expand them, and to our surprise, after several weeks of culture in the presence of puromycin, when we examined the distribution of class I molecules using immunofluorescence microscopy, the cells had regained their ability to divert class I MHC molecules to the lysosomal compartment. We then repeated the experiment, taking careful account of surface class I MHC molecules after AP-1 or AP-3 shRNA expression. Four days after infection with μ3 shRNA, class I MHC molecules redistributed from lysosomes to the cell surface (Figure 3, panel c, and Figure 7, panel a). By 8 days, however, the phenotype began to revert: the cells distributed bimodally, reflecting a population of cells that almost completely lacked class I MHC molecules on their surface, and a population of cells that had restored surface expression of surface class I MHC molecules (Figure 7, panel b). The population of cells remained heterogeneous after 35 days (Figure 7, panel c), but by 50 days, the entire population of μ3 depleted cells resembled U21 cells before depletion of the μ subunit (Figure 7, panel d). μ1 deficient cells behaved similarly; when we generated stably-expressing μ1 shRNAs, after 50 days, class I MHC molecules returned to the cell surface (data not shown).

We first assumed that the shRNA was ineffective over time, and that protein levels of μ1 and μ3 had returned to normal. However, when we examined levels of μ3 in the μ3-depleted U21 cells, μ3 levels remained reduced by 90% (compare Figure 7e, lanes 1 and 2 to Figure 3b, lanes 1 and 2). To explain this phenomenon, we considered the possibility that other AP subunits or other proteins might compensate for absent μ3. For example, there are two AP-1μ proteins, denoted μ1A and μ1B. μ1B expression is confined exclusively to polarized cell types [25], [39]. Although μ1B should not be expressed in our U373 astrocytoma cells, we thought it possible that expression of this subunit might have become somehow upregulated in the absence of μ1A, and that upregulated μ1B might be able to substitute for μ1A. We therefore performed immunoblot analysis with an μ1B-specific antiserum, but we were unable to detect the presence of μ1B in the U373-U21 cells, even when expressing μ1A shRNA (data not shown).

We also considered the possibility that enough of the residual μ subunits existed to allow sorting of protein X, albeit slowly, such that after 50 days, protein X and U21 could reroute the bulk of class I MHC molecules to the lysosomal compartment. To examine this possibility, we reasoned that we should be able to deplete the remaining μ subunits in these stable cell lines, and if residual μ subunit molecules were responsible for the eventual rerouting of class I molecules, further depletion should result in the return of class I MHC molecules to the cell surface. We chose to transiently reduce any remaining μ3 *via* transfection of a small interfering RNA (siRNA) that hybridized to a site distinct from the μ3 shRNA used for generating the stable μ3-depleted cells. After μ3 siRNA transfection, we examined lysates for μ3 protein expression and observed depletion of residual μ3 to non-detectable levels (Figure 7e, lane 3). After siRNA transfection, class I MHC molecules once again returned to the cell surface (Figure 7, compare panels f and g). We obtained similar results with AP-1; when we transfected μ1 siRNA to reduce residual levels of μ1 in cells stably expressing μ1 shRNA, class I MHC molecules returned to the cell surface (data not shown). These results suggested that the residual μ3 or μ1 had formed enough functional AP-3 or AP-1 complexes to sort protein X to the lysosomal pathway, after time in culture. Thus, whereas transient depletion of adaptor complexes resulted in a dramatic shift in class I MHC localization, chronic depletion of the complexes somehow allowed the cells time to sort U21 and class I molecules to lysosomes.

### Constitutive vs. Transient Depletion of AP Subunits

The MCMV m06 gene product, gp48, also reroutes class I MHC molecules to the lysosomal compartment. Unlike U21, MCMV gp48 contains a canonical di-leucine sorting signal in its cytoplasmic tail that, when mutated, impairs the ability of gp48 to reroute class I MHC molecules [20]. Since MCMV is a murine herpesvirus, Reusch, *et al.* were able to take advantage of MEFs derived from AP-1 and AP-3 knockout mice to assess the role of adaptor complexes in gp48-mediated trafficking of class I MHC molecules to lysosomes. In mouse embryonic fibroblast (MEF) cells generated from AP-1μ^−/−^, AP-3δ^−/−^, or doubly-deficient AP-1μ^−/−^/AP-3δ^−/−^ mice, Reusch *et al.* demonstrated the importance of both of these AP complexes in gp48-mediated trafficking of class I MHC molecules. Interestingly, when Reusch *et al.* expressed gp48 in these MEFs, they saw no effect of the absence of either AP-1μ or AP-3δ on surface class I MHC expression. It was not until they examined the ability of m06 to divert class I molecules in MEFs made from doubly-deficient AP-1μ ^−/−^/AP-3δ^−/−^ mice that the role for these adaptor complexes was clear; m06 was unable to effect the removal of class I molecules from the cell surface in the AP-1μ^−/−^/AP-3δ^−/−^ cells [20].

Because long-term shRNA-mediated depletion of AP-1 and AP-3 μ subunits from U21-expressing cells resulted in the eventual localization of class I molecules in lysosomes, we were unsure whether the ability of U21 to affect lysosomal sorting would depend upon whether adaptor complexes were acutely reduced, or chronically absent. For this reason, we were hesitant to draw a parallel between our results and those seen by Reusch, et al., for MCMV gp48, in which experiments to examine gp48’s effect upon class I MHC molecules in the absence of adaptor complexes were performed in cells derived from AP-1- and AP-3-deficient mice. We therefore decided to express MCMV gp48 in U373 cells and determine whether acute siRNA-mediated knockdown of individual AP subunits would affect gp48’s ability to reroute class I MHC molecules.

**Figure 8:**
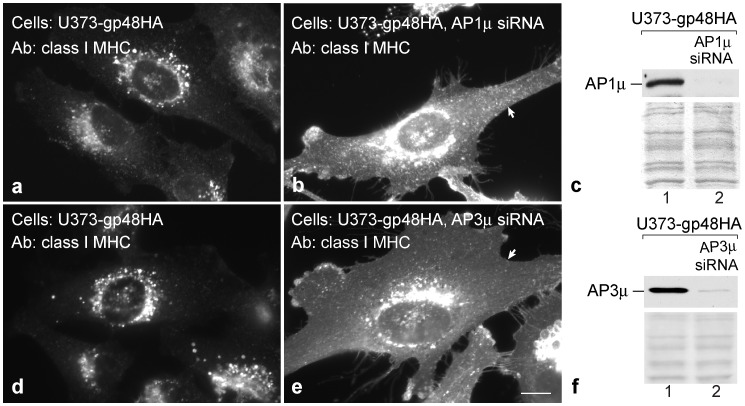
MCMV gp48-mediated trafficking of class I MHC molecules is affected by depletion of AP-1μ or AP-3μ subunits. Immunofluorescence analysis of class I MHC molecules in cells expressing gp48HA, − (a) and + (b) AP-1μ siRNA, and - (d) and + (e) AP-3μ siRNA, as indicated. Arrows in panels b and e point to the plasma membrane. Scale bar = 10 µm. c and f) Immunoblot analysis of AP-1μ or AP-3μ from lysates of U373-gp48HA cells −/+ treatment with AP-1μ (c) or AP-3μ (f) siRNA, as indicated. The Ponceau S stained nitrocellulose is shown beneath the immunoblots as a loading control.

For these experiments, we first ascertained that MCMV gp48 would function similarly in human cells to affect the localization of human class I MHC molecules. We expressed gp48 and gp48 containing a C-terminal HA tag in U373 cells, and then examined the localization of class I MHC molecules. As shown in Figure 1d, class I MHC molecules are located largely on the cell surface of control U373 cells. When we examined endogenous class I MHC molecules in U373 cells expressing either gp48 or gp48-HA, class I MHC molecules appeared in a punctate perinuclear distribution very similar to class I molecules in cells expressing HHV-7 U21 (Figure 8, panels a and d, untagged gp48 not shown), and similar to the pattern of class I molecules in murine cells expressing gp48 [11], suggesting that, like U21, gp48 can also reroute both human and murine class I MHC molecules to lysosomes [28]. We then determined whether transient siRNA-mediated reduction of μ1 or μ3 could affect gp48’s ability to reroute class I molecules. Immunoblot analysis confirmed efficient (97% and 90%) depletion of the detectable μ1 and μ3, respectively (Figure 8, panels c and f). While expression of gp48 in individually deficient AP-1- or AP-3- MEFs had no effect upon class I localization [20], transient RNAi-mediated reduction of either μ1 or μ3 resulted in dramatic relocalization of class I MHC molecules to the cell surface (Figure 8, panels b and e). These results illustrate the potential for phenotypic difference when examining cell lines constitutively deficient in AP complexes *versus* cells after more acute RNAi-mediated knockdown.

## Discussion

The process of trafficking of integral membrane proteins to the lysosomal pathway is generally mediated by proteins that recognize various cytoplasmic sorting signals. The lamp proteins, for example, contain a tyrosine-based sorting signal in their cytoplasmic tails that are recognized by the clathrin adaptor proteins AP-1 and AP-3. If the tyrosine-based sorting signal in lamp-1 is mutated, rather than traffic via the direct pathway to lysosomes, lamp-1 travels via the indirect route, making appearances at the plasma membrane and in endosomes while *en route* to the lysosomal compartment [40]. Since endogenous lamp-1 ultimately accumulates in lysosomes regardless of adaptor protein complex depletion, the necessity of adaptors in the efficient trafficking of endogenous lysosomal membrane proteins has been evaluated by examining the appearance of lysosomal proteins at the cell surface. In cells lacking AP-3, for example, lysosomal proteins such as CD63, lamp1, and lamp2 make an appearance on the plasma membrane and in endosomes, suggesting that when adaptor complex-mediated trafficking is impaired, lysosomal proteins can travel through a plasma membrane-endosome recycling pathway while *en route* to lysosomes [41]–[44].

While the role for AP-3 in trafficking of membrane proteins to lysosomes is fairly well-established in cells lacking AP-3, the role of AP-1 in trafficking the lamp proteins to lysosomes is less clear. In μ1A-adaptin-deficient murine fibroblasts, no alteration of trafficking of lamp-1 to lysosomes was observed, suggesting that AP-1 is not essential for lamp localization [32]. Chapuy *et al*., point out that the lamp-1 sorting signal displays similar binding affinities for μ1, μ2, and μ3 adaptor subunits, and suggest that perhaps the low degree of specificity for these adaptor complexes may allow lamp-1 to travel to lysosomes via any of these pathways, since they all seem to lead to lysosomes [45]. Even in doubly-deficient AP-1A^def^/AP-3A^def^ murine cells, lamp1 localization was normal, suggesting that lamp-1 can effectively use the indirect, AP-2 mediated pathway [20]. An RNAi-mediated approach to depleting stores of specific AP complex subunits led to a different conclusion: Janvier *et al*., when examining lamp localization after using siRNA to deplete the μ subunits of AP-1, AP-2, and AP-3, demonstrated that AP-2 depletion resulted in the most striking redistribution of lamp to the plasma membrane, suggesting that lamp normally traffics *via* the plasma membrane *en route* to lysosomes [30].

In constructing a model for U21-mediated trafficking of class I MHC molecules to lysosomes, the simplest is one exemplified by the MCMV m06 immunoevasin [11]. MCMV m06 contains a di-leucine sorting signal in its cytoplasmic tail that requires functional AP-1 and AP-3 clathrin adaptor complexes. These adaptor complexes mediate sorting of the m06-class I MHC complex to the lysosomal compartment [20]. We initially postulated a similar mechanism for U21, but instead, we found that U21 can affect the trafficking of class I molecules even when its cytoplasmic tail is deleted [8], and even when the U21 molecule is in a soluble secreted form [22]. Thus, if the lysosomal sorting signal utilized by U21 is cytoplasmic, we could envision two possible models: either the sorting signal exists within cytoplasmic tail of the class I molecule, or another cellular protein associates with the U21/class I complex that contains the information necessary to reroute the class I MHC-U21 complexes. Since U21 can also reroute a tailless version of HLA-A2 class I MHC molecules to the lysosomal compartment [22]), we were left with the idea that a cellular protein X, that contains the information necessary to reroute class I MHC/U21 complexes, must associate with the U21/class I MHC complex.

With this model in mind, we began with the hypothesis that U21 utilizes a cellular protein that contains lysosomal sorting information. If so, we reasoned that the sorting signal is likely to be cytoplasmic, and, just as for the viral immunoevasins MCMV m06 and HIV Nef, the sorting signal-bearing protein might ultimately utilize clathrin adaptor protein complexes as mediators of this sorting event [11], [20], [35], [46]. It follows that knockdown of the clathrin adaptor protein complexes should affect U21’s ability to divert class I MHC molecules to lysosomes.

While AP-1 or AP-1/AP-3 have been shown to mediate lysosomal trafficking of class I molecules by HIV Nef and MCMV m06 [20], [35], we were still unsure whether U21 and its client class I MHC molecules traveled *via* the direct or indirect route to lysosomes. Since AP-2 mediates internalization from the cell surface, involvement of AP-2 in U21-mediated sorting of class I MHC molecules to lysosomes would suggest that U21 uses the indirect pathway. We therefore depleted clathrin adaptor complexes, AP-1, AP-2, AP-3 and AP-4. Depletion of AP-1 and AP-3, but not AP-2 or AP-4, affects U21-mediated diversion of class I molecules, suggesting that U21 and protein X travel via the direct clathrin-mediated pathway to the lysosomal compartment from the TGN. Interestingly, when we depleted either AP-1 or AP3, the cell surface expression of class I MHC molecules increased even beyond the levels of class I molecules expressed in control U373 cells (Figures 2 and 3). We speculate that, in the absence of the adaptors responsible for sorting from late endosomes to lysosomes, that plasma membrane protein turnover and trafficking in general is slowed, resulting in delayed turnover of surface class I MHC molecules. AP-1 and AP-3 involvement in U21-mediated diversion of class I molecules to the lysosomal compartment also suggests that unlike MCMV m06, which recruits the AP-1 complexes directly, HHV-7 U21 utilizes a cellular protein X for the recruitment of AP-1 and AP-3. This idea is underscored by our finding that AP-1 depletion affects U21-mediated rerouting of class I molecules even when U21 lacks its cytoplasmic tail.

Although our experiments were designed to contribute to our understanding of the U21-mediated trafficking of class I MHC molecules, during the course of these studies, we made two separate observations that provide cautionary information regarding the interpretation of experiments performed in cells chronically depleted of AP subunits. First, our stable μ1A and μ3 shRNA-expressing cells reverted their phenotypes after several weeks in culture; this phenotype reversion appeared not to be the result of selection for cells that had less-severe depletion of μ1A or μ3, since steady-state levels of the μ subunits remained low. Instead, the ability to reroute class I molecules to lysosomes after μ1A or μ3 depletion appeared to be the result of the remaining 2–10% of the μ subunits remaining in the cells after shRNA-mediated depletion, since subsequent siRNA-mediated knockdown of the remaining μ subunits in these cells successfully restored the original shRNA-mediated effects (Figure 7). These experiments suggest that, given time, a small fraction of the normal population of adaptor protein complexes can adapt to carry out U21-mediated sorting of class I MHC molecules, or that a population of cells was selected for that could somehow utilize a very small pool of μ subunits to reroute class I molecules.

Second, murine cells deficient in either AP-1μ or AP-3δ had no immediate effect upon gp48’s ability to reduce surface expression of class I MHC molecules [20], yet our experiments examining the localization of class I MHC molecules in gp48 cells transiently depleted of μ1A or μ3 showed striking effect of depletion of single μ1A or μ3 subunits (Figure 8). We are therefore led to suggest the possibility that murine AP-1- and AP-3-deficient fibroblasts have somehow compensated for the lack of adaptor complex subunits during their long-term passage. These experiments underscore the thoroughness of Reusch, *et al.* in their interpretation of the involvement of AP-1 and AP-3 in the trafficking of m06 and class I molecules to lysosomes. Had these authors not taken the extra care to examine the ability of m06 to reduce the presence of class I MHC molecules on the surface of AP-1/AP-3 doubly-deficient cells, they may have erroneously concluded neither AP-1 nor AP-3 participate in the trafficking of m06 and class I MHC molecules. These results may have broader implications for the interpretation of chronic silencing experiments; for essential cellular processes, cells may likely find ways to circumvent the loss of a particular protein in order to survive.

Our results demonstrate that like di-leucine-containing MCMV gp48, HHV-7 U21 utilizes both AP-1 and AP-3 to reroute class I MHC molecules to the lysosomal compartment. Since the cytoplasmic tail of U21 is not necessary for its trafficking of class I molecules to lysosomes, the requirement for AP-1 and AP-3 suggest that the sorting signal involved in U21-mediated trafficking to lysosomes is likely contained within an as-yet unknown cellular protein. We have searched for cellular proteins that associate with the U21/class I MHC complex without success; immunoprecipitations of U21 and class I MHC molecules have yielded no other obvious co-precipitating proteins involved in trafficking [7], [8], [22], [36]. That this cellular binding protein is not seen in immunoprecipitation experiments is not surprising; it is possible that the cellular protein associates with the U21/class I MHC complex transiently, mediating only TGN-late endosomal sorting (similar to the mannose 6-phosphate receptor). It is also possible that the cellular protein's association with the U21/class I MHC complex is not easily preserved upon detergent lysis, even under gentle solubilization conditions. Further efforts to identify this cellular protein are underway.
